# Quality assessment of “naturally occurring” high-percentage L-dopa commercial products proposed as dietary supplements on the Internet: from labeling to analytical findings

**DOI:** 10.3389/fchem.2025.1597784

**Published:** 2025-10-15

**Authors:** Federica Aureli, Maria Cristina Gaudiano, Mariangela Raimondo, Alessandro Maccelli, Domenico Di Giorgio, Marta Gramazio, Anna Borioni, Monica Bartolomei

**Affiliations:** 1 Chemical Medicines Unit, National Centre for the Control and Evaluation of Medicines, Istituto Superiore di Sanità, Rome, Italy; 2 Inspection and Certification Department, AIFA - Italian Medicines Agency, Rome, Italy

**Keywords:** mass spectrometry, nuclear magnetic resonance, L-dopa, dietary supplements, nootropic, plant extract, Parkinson’s disease

## Abstract

**Introduction:**

Levodopa (L-dihydroxyphenylalanine or L-dopa) is a precursor of the neurotransmitter dopamine and is used in Parkinson’s disease therapy. L-dopa dietary supplements are widely marketed as brain support. Among the L-dopa products claiming to contain botanical extracts, those from *Mucuna pruriens* are the most frequently offered on the Internet. The natural percentage of L-dopa in *M. pruriens* seeds or leaves varies from 1% to 7%, but extracts standardized at higher percentages of L-dopa are also available.

**Methods:**

Four L-dopa products marketed as dietary supplements were purchased online and analyzed for labeling accuracy. The identification of L-dopa and the detection of undeclared pharmaceutical or nootropic ingredients were carried out via mass spectrometry (LC-MS Q-TOF). Nuclear magnetic resonance (NMR) spectroscopy was used to confirm the presence of L-dopa and quantify it. Furthermore, a Google Trends analysis was conducted to study interest in the terms “levodopa” and “brain supplements” and their Italian equivalents, over the last 10 years in Italy and globally.

**Results:**

Visual inspection of labeling revealed that the extract of *M. pruriens*, which is not allowed in dietary supplements in Italy, was listed on three products, while the extract of *Vicia faba* was declared in one sample. Some labeling concerning the dosage of L-dopa was ambiguous. LC-MS and NMR analyses confirmed the presence of L-dopa in all the samples. No undeclared active pharmaceutical or nootropic ingredients were detected. The amount of L-dopa in the capsules was found to match the labeled dosage in some samples, but others were either overdosed or underdosed. Trend analysis indicated increasing interest in the terms “levodopa” and “brain supplements” both in Italy and worldwide.

**Discussion:**

The obtained results showed evidence of potential risks related to consuming dietary supplements purchased online containing high-dose L-dopa. These risks arise from the inclusion of unauthorized botanical extracts, unclear labeling, and inconsistencies between labeled and actual dosages. Given the observed increasing public interest in levodopa, these findings highlight the need to control this market and inform consumers and physicians about the risks of purchasing unauthorized online products.

## 1 Introduction

Levodopa (L-dihydroxyphenylalanine or L-dopa), belonging to the class of catecholamines, was discovered in the late 1950s to early 1960s and remains one of the most common therapies for treating motor symptoms in Parkinson’s disease to this day (Hornykiewicz, 2017). As a precursor of the neurotransmitter dopamine, it is absorbed from the intestinal tract and crosses the blood–brain barrier via active transporters to reach the brain, where it is converted into dopamine (Rezak, 2007). The pharmacological efficacy of L-dopa decreases after a certain period of intake, which often requires the adjustment of therapies. The reported side effects of L-dopa include non-motor adverse effects such as i) nausea, vomiting, and dry mouth; ii) orthostatic hypotension, dizziness, headache, weakness, and somnolence; iii) cardiac dysrhythmias; and iv) hallucinations, chest pain, and depression and the motor adverse effects include motor fluctuations (on–off phenomenon) and dyskinesias (Bhushan et al., 2023; Mouchaileh and Hughes, 2020). To decrease the side effects, L-dopa is co-administered with carbidopa, entacapone, or benserazide (Katzung et al., 2024). The use of L-dopa preparations as nootropics or “brain support” is increasing due to the general increase in interest in products that enhance cognitive performance and improve mental focus and memory (Vanhee et al., 2025). Moreover, in bodybuilding forums, L-dopa dietary supplements are recommended alone or in combination with other nootropics to achieve dopaminergic stimulation or with 19-nor derivatives, such as trenbolone, to counteract dopamine depletion.

L-dopa can also be found in natural sources, particularly in plants of the Fabaceae family (e.g., beans, peas, faba beans, lentils, peanuts, and soybeans) (Boelens Keun et al., 2021), with Mucuna pruriens and its derived products being the most commonly promoted online for natural L-dopa supplementation. Different varieties of plants and individual parts of plant matrices (e.g., seeds and leaves) can contain various amounts of L-dopa. The natural concentration of L-dopa in *M. pruriens* ranges from less than 1% to 7% (Adebowale et al., 2005; Nishihara et al., 2005; Pulikkalpura et al., 2015; Soares et al., 2014). Faba bean shows a lower content of L-dopa on average, with quantities of < 1% in the leaf, flower, seeds, and pod extracts, which reach up to 3% in pod valves (Tesoro et al., 2024; Topal and Bozoğlu, 2016). Moreover, commercially available products marketed as vegetal preparations, also known as botanicals, or dietary supplements are sold online with high levels of L-dopa (15%, 25%, or practically pure products), indicating that they result from intensive extraction and concentration procedures or the addition of chemically synthesized pure active substance (Tesoro et al., 2022). In the European Union (EU), botanicals and botanical preparations can enter the market as food supplements or herbal medicinal products, provided they comply with the requirements set out in the respective food and medicinal legislation. The EU does not have a centralized authorization procedure for the use of botanicals and their derived preparations in food; instead, each Member State has developed national provisions to complement the general food regulatory framework. In Italy, the use of plant extracts and preparations in food supplements is currently regulated by the Ministerial Decree of 10 August 2018, which, among other provisions, sets out a list of permitted plants, either as a whole or as specific parts (seeds, leaves, etc.) (Ministero della Salute, 2018). This list is only relevant to plants/parts of plants and their derivatives (e.g., extracts or other preparations) with a history of significant food consumption before 1997; substances, preparations, and extracts obtained from the listed plants but without the cited history of consumption are considered novel foods (food that had not been consumed to a significant degree by humans in the EU before 15 May 1997) under Regulation (EU) 2015/2283 and, therefore, cannot be used prior to EU authorization.

Irrespective of their classifications and use, the illicit trade of commercial products containing undeclared/unauthorized pharmaceutical ingredients, nootropic agents, and unauthorized novel food is still increasing, and a variety of unauthorized products can be found on the Internet (Gaudiano et al., 2022; Gaudiano et al, 2024a; Gaudiano et al, 2024b; Vanhee et al., 2025). The safety profile of illegal products is unknown, posing a tangible health risk to consumers.

The identification and quantification of L-dopa in botanicals and botanical preparations proposed as dietary supplements is a crucial aspect in their quality assessment and for the verification of possible adulteration with other nootropic active ingredients that could be fraudulently added to these products. Different high-performance liquid chromatography (HPLC) and liquid chromatography–mass spectrometry (LC–MS) methods have been validated to quantify L-dopa in plant matrices (Kaced et al., 2024; Tesoro et al., 2022; Tesoro et al., 2023; Vora et al., 2018; Yumoto et al., 2022) and commercial *M. pruriens* products (Hasegawa et al., 2011; Soumyanath et al., 2018). Simpler methods such as high-performance thin-layer chromatography (HPTLC) have also been developed for the analysis of L-dopa in *M. pruriens* seed extract and its formulations (Aware et al., 2017; Modi et al., 2008; Pulikkalpura et al., 2015). Nuclear magnetic resonance (NMR) has been extensively used in the field of botanical health products analysis and quality control (Zhao et al., 2022), and quantitative NMR (q-NMR) has been applied for the analysis of L-dopa in *M. pruriens* seeds (Fernandez-Pastor et al., 2019).

Taking into account that L-dopa is an active ingredient with a consolidated use for the treatment of Parkinson’s disease, the aim of the present study was to assess the quality of products proposed as dietary supplements with declared L-dopa content and purchased via the Internet from retail websites accessible from Italy. Samples were evaluated for labeling and analyzed using an LC–MS screening method and NMR to identify L-dopa and assess possible adulteration with other nootropic and pharmaceutical active ingredients. Quantification of L-dopa in the products was performed by q-NMR to evaluate whether the actual amount of L-dopa complied with what was declared and whether it was compatible with the quantity derived from a natural extract. This research targets the first country-wide investigation of the illicit L-dopa online market in Italy. Finally, a study was conducted on web search trends for the terms “levodopa” and “brain supplements” and their Italian equivalents, comparing the situation in Italy with global trends.

## 2 Materials and methods

### 2.1 Selection of the retail websites

Websites accessible from Italy selling L-dopa “dietary supplements” were selected using the Google search engine using the Italian terms *“acquista integratori L-dopa in Italia”* (buy L-dopa dietary supplements in Italy) as the keywords. Websites based in Europe were prioritized for product purchases. Retail websites were chosen based on sale conditions: the possibility to pay by prepaid credit card and ship the product to Italy. These websites reported information in the Italian language; the IP addresses, investigated using the site https://www.iplocation.net/ip-lookup, were located in Italy (two sites), the Czech Republic (one site), and Germany (one site). The purchases were made by the Italian Medicines Agency.

The retail websites were selected if they explicitly mentioned products showing nootropic activities with claims such as “an exceptional extract that helps maintain a healthy nervous system,” “a leading compound in Ayurvedic medicine,” “guaranteed without synthetic additives,” and “precursor of the neurotransmitter dopamine.”

### 2.2 Samples and visual analysis

Four capsule products claiming to contain L-dopa from botanical extracts were purchased from four different manufacturers: three samples were labeled as naturally extracted from *M. pruriens*, and one sample was from *Vicia fab*a. To avoid being traced as an official laboratory of control, the samples were sent to a private address. The samples were photographed and subjected to visual analysis of the packaging and labeling before instrumental analysis.

### 2.3 Trend of interest

The Italian and global trends of interest for the terms *“brain supplements”* and “*levodopa”* for “all categories” in the period from 01/12/2015 to 31/01/2025 were analyzed using the Google Trends application. Results are expressed as the relative normalized search volume numbers and are presented on a scale from 0 to 100, where 100 represents a maximum search interest at a given time and place. This tool allows investigating the popularity of a selected term in the specified period of time and region. The numbers reflect how many searches were performed for a particular term relative to the total number of searches performed using Google over time. Each point on the graph is divided by the highest point, which is conventionally set at 100 (Gaudiano et al, 2024a; Google, 2023). “*Levodopa*” was used in the searches for both the “World” and “Italy” regions, considering that the term is identical in English and Italian. Moreover, the term *“brain supplements”* was used for the “World,” while the combined search terms *“integratori memoria + integratori concentrazione*” were used for Italy as these are used in everyday language to refer to common brain supplements.

### 2.4 LC–MS analysis

The identification analysis was performed using the same screening method reported elsewhere (Gaudiano et al, 2024a). In brief, a liquid chromatograph (Model 1290 from Agilent Technologies, Santa Clara, California) coupled with a high-resolution quadrupole time-of-flight mass spectrometer (Model 6520 from Agilent Technologies), equipped with a dual electrospray ionization (dual ESI) source working in both positive and negative mode, was used. All the operations, acquisition, and analysis of data were controlled using Agilent MassHunter Acquisition software version B.06.01 and processed using MassHunter Qualitative Analysis software version B.07.00. Each sample was analyzed in positive mode over the range of *m/z* = 50–1,200 in the extended dynamic range (2 GHz). The same condition was applied to negative mode. Accurate mass measurements were obtained by reference mass correction using masses at *m/z* 121.0509 (purine) and 922.0098 (hexakis (1H, 1H, 3H-tetrafluoropropoxy) phosphazene or HP-921) in positive ion mode and 112.9856 (trifluoroacetic anion) and 1,033.9881 (HP-921) in negative ion mode. All solvents and reagents were of LC–MS grade (Merck KGaA, Darmstadt, Germany). The reference standard L-dopa was purchased from Sigma-Aldrich (St Louis, MO, United States).

Chromatographic separation was carried out on a ZORBAX Extend-C18 Column (2.1 × 50 mm, 1.8 µm particle size) using a linear gradient elution from a high concentration of water (95:5 water/acetonitrile containing 0.1% formic acid) to a high concentration of organic solvent (5:95 water/acetonitrile containing 0.1% formic acid) over 15 minutes. The flow rate was set at 0.4 mL/min, and the injection volume was 1 μL. The column temperature was 35 °C, and the autosampler was thermostated at 10 °C. Mass spectrometer parameters were as follows: fragmentor, 100 V; nitrogen temperature, 300 °C; drying gas, 10 L/min; nebulizer, 40 psig; and VCap, 4,000 V. The high resolution of the instrument allowed for accurate measurement of ionic masses in MS mode and enabled the calculation of the chemical formulas of the molecules contained in the samples. Target MS/MS studies were performed by isolating [M+H]^+^ or [M-H]^-^ ions. The major fragments were obtained for the protonated/deprotonated molecule and compared with those produced for the standard under identical conditions. Fragmentation patterns were obtained at 20 V of collision energy offset. Auto MS/MS analysis was used to screen for undeclared active pharmaceutical substances using the Agilent *MassHunter Forensics and Toxicology Personal Compound Database and Library* (*ForTox PCDL* B.07.01). The exact mass and ion fragmentation pattern of each detected compound were then compared with the PCDL library, which contains > 9,000 exact ion masses and > 3,000 ion fragmentation patterns, including common nootropic substances and active pharmaceutical ingredients.

For each product, the powder contained in a capsule was transferred to 50 mL or 100 mL volumetric flasks and dissolved with methanol. The solution was sonicated for 10 minutes and magnetically stirred for 1 hour. The solution was then diluted at a 1:10 ratio in acetonitrile/water (1:1 v/v) containing 0.1% formic acid and filtered through 0.22 µm PTFE filters in order to achieve an estimated L-dopa concentration of approximately 0.1 μg/μL for injection into the LC–MS system, based on the labeled content.

### 2.5 NMR analysis

The NMR experiments were performed on a Bruker AVANCE Neo 600 MHz Spectrometer (Bruker BioSpin GmbH, Germany), operating at 14.09 T and equipped with a Bruker iProbe 5 mm SmartProbe. The NMR data were collected and processed using TOPSPIN 4.1.3 and IconNMR software (Bruker BioSpin, United States). The samples were analyzed to confirm the presence of L-dopa in the capsules and determine its content.

#### 2.5.1 Identification

An aliquot of the capsules’ content corresponding to approximately one-quarter of the reported L-dopa content was dissolved in 1 mL of methanol-d4 (Sigma-Aldrich). The samples were vortexed for 1 minute and allowed to settle for 10 min. A measure of 0.6 mL of the supernatant was transferred into the NMR tube.

Identification was carried out using one- and two-dimensional Bruker’s pulse programs: 1H-(zg), ^1^H–^1^H total correlation spectroscopy (TOCSY), ^1^H–^13^C heteronuclear single quantum coherence (HSQC, hsqcedetgpsisp2.3), and heteronuclear multiple bond correlation (HMBC, hmbcetgpl3nd).

#### 2.5.2 Quantitative analysis

The choice of the internal standard/solvent system for q-NMR was based on the solubility of L-dopa and the standard and the good separation of their respective NMR signals in the spectra. L-dopa was extracted using a mixture of deuterium oxide (D 99.6%) and acetic acid-d4 (Cambridge Isotope Laboratories) according to the solubility data reported in the literature (Polanowska et al., 2019). Maleic acid (TraceCERT^®^ Maleic Acid, Sigma-Aldrich, Milan, Italy), displaying a ^1^H-NMR signal at 6.1 parts per million (ppm), was chosen as the internal reference standard. This compound was chosen as the internal standard because of the sharp singlet signal in a region of the NMR spectrum with no other signals and for its insensitivity to the matrix effects, solubility, and stability in a solution. The capsule powder from each sample was dissolved in acetic acid-d4/D_2_O (20:80, v/v) at concentrations ranging from 5 mg/mL to 20 mg/mL of the capsule content, corresponding to approximately 0.5 mg/mL–13 mg/mL of labeled L-dopa content. The mixtures were vortexed for 1 min, sonicated for 15 min, and centrifuged at 10^4^ rpm for 15 min. A volume of 0.7 mL of each solution was transferred into a vial containing 5 mg of maleic acid and vortexed for 2 min. Finally, the solutions were transferred to the NMR tubes for analysis.

q-NMR analysis was performed using the Bruker pulse program (zg) with the following settings: 32 scans, size of FID (TD) = 64K, spectral width (SW) = 25 ppm centered at (O1P) 4.70 ppm, and target probe temperature (TE) = 298.0 K. In addition, good signal intensity and recovery were ensured by the determination of the relaxation time (T1) and the calibration of the 90° pulse width (p1) prior to each analysis. The delay time D1, set at 7*T1 (60 s), allowed for 99.9% signal recovery. All spectra were apodized with an exponential function at 0.3 Hz, processed with zero filling (128 K), and subjected to phase adjustment and baseline correction. If necessary, manual phasing and baseline correction in selected spectral ranges were applied. Visual examination of the 2D NMR experiments (TOCSY, HSQC, and HMBC) confirmed that no interfering signals were present under the signals of L-dopa signals selected for quantification.

The reliability of the results was first based on the control of the equipment performance and the compliance to the requirements laid down in the European Pharmacopoeia (Ph. Eur.) and the ISO 17025:2017 system. At each analytical session, sensitivity (by checking the S/N), the homogeneity of the magnetic field (by the 1H-line shape test), and the 90° pulse length (related to the reproducibility of the measures) were assessed according to the manufacturer’s acceptance criteria, and they support the suitability of the equipment for qNMRs in the applied range.

According to the Ph. Eur. monograph nuclear magnetic resonance spectrometry, a signal-to-noise ratio (S/N) of at least 150 allows peak integrations with a standard deviation of less than 1%. The S/N on the integrated signal of L-dopa in all the lower-concentration samples was higher than 150. As also required by the Ph. Eur., only signals with at least five digital points above the half-height were considered for quantification. This number of points ensures that the integrated signals are well-defined. Accuracy is also dependent on the spectra acquisition settings, which must ensure the full recovery of the integrated signals. This was achieved by a suitably long interscan delay (D1 = 60 s), which was experimentally determined for each sample. The accuracy also depends on the portion of the integration region. The NMR signals have a Lorentzian line shape that decays infinitely with a steepness value dependent on the full width at half height (FWHH) of each signal. Therefore, full signal integration is not realistic. Thus, the integration range of the sample and the reference standard signals was carefully selected. The FWHH of the L-dopa and maleic acid signals was measured, and the proportional regions corresponding to equal areas of the signals were integrated.

Once all the NMR experimental parameters were set correctly, the only factor that could affect the accuracy of the results was the efficiency of the L-dopa extraction from the matrix. This issue was addressed by checking the linearity of the results, as reported below. After preliminary experiments with methanol, which produced unsatisfactory results, the powder in the capsules was extracted using a 20% acetic acid-d4/D_2_O solution. This approach was highly effective in distinguishing L-dopa from the other components.

## 3 Results

### 3.1 Visual analysis

**FIGURE 1:**
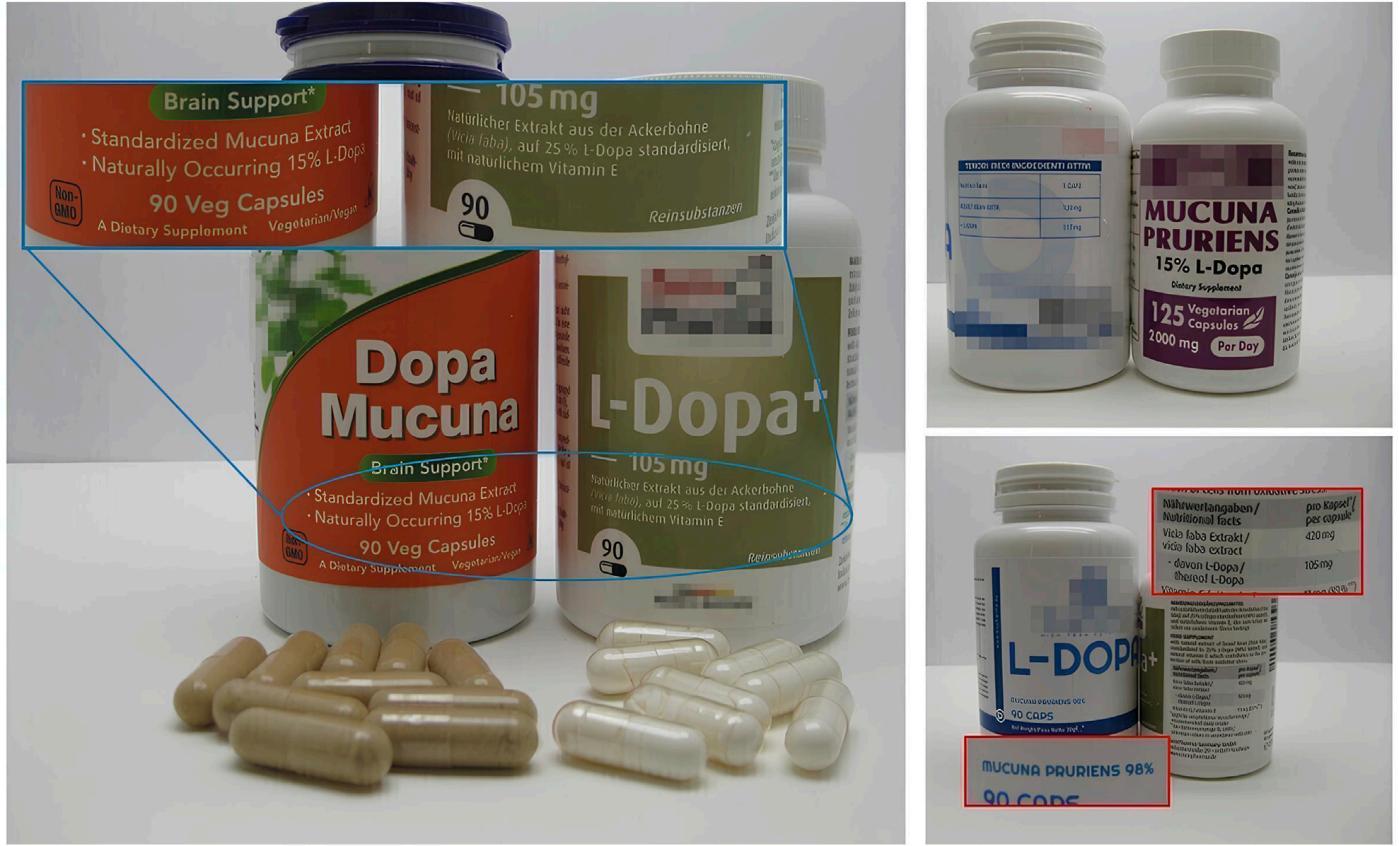
Photographs of the samples with details of the reported contents on the labels.

The results of the visual analysis of the samples are reported in Table 1. All samples, labeled as dietary supplements, were in capsules form and contained L-dopa extracts standardized from 15% to 98% (see images in Figure 1). The content of L-dopa for capsules was not clearly indicated in all the samples; in two samples (samples #3 and #4), the content was calculated on the basis of the reported percentage of L-dopa in the extract and the serving size. The labeled L-dopa content per serving ranged from 105 mg to 300 mg, with the last quantity exceeding the recommended initial daily dose for Parkinson’s disease treatment (200 mg in combination with other active substances). In one case (sample #3), the use as “brain support” was explicitly reported. The manufacturer’s name was absent in one case (in sample #4, only the country was reported). In one case only, labeling was provided also in Italian language. Samples #1, #3, and #4 did not report the part of the plants used to produce the extract. In three samples, the plant source of the extract was identified as *M. pruriens*. The content per capsule of the vegetal extract was reported only for three samples.

**TABLE 1:** Findings of the visual inspection of labeling.

Sample #	1	2	3	4
Labeled vegetal extract content per cps^a^	420 mg	N.R^b^	400 mg	400 mg
Vegetal extract name	*Vicia faba*	*Mucuna pruriens*	*Mucuna pruriens*	*Mucuna pruriens*
Labeled % of L-dopa in the extract	25% (natural extract)	98%	15% (naturally occurring)	15%
Labeled L-dopa content per cps	105 mg	117 mg	N.R. (explicitly) ^c^	N.R. (explicitly) ^d^
Number of cps per serving	1 cps/die	1 cps/die	2 cps/die	5 cps/die
Labeled L-dopa content per serving	105 mg	117 mg	120 mg	N.R.^e^ (explicitly) ^e^
Labeled ingredients	*Broad bean extract (25% L-dopa), hydroxypropyl methyl cellulose (capsule), and D-a-tocopherol acetate (vitamin E)*	*Velvet bean extract (Mucuna pruriens) standardized 98% of L-dopa, anticaking: magnesium salts of fatty acids, silicon dioxide, and gelatine (capsule)*	*Amount per serving* *Mucuna extract (Mucuna* spp.*) (seed) (min. 15% L-dopa, naturally occurring) 800 mg* *-------------------------* *L-dopa 120 mg* *Other ingredients: hypromellose (cellulose capsule), microcrystalline cellulose, and stearic acid (vegetable source)*	*Each vegetarian capsule contains 400 mg of the Mucuna pruriens extract standardized to 15% L-dopa* *Other ingredients: acacia gum and white rice flour*
Reported pharmacological activity	N.R.	N.R.	Brain support	N.R.
Reported manufacturing name and country	Yes (Germany)	Yes (SLO)	Yes (USA)	Only reported the country (United States) and EU distributor name
Reported batch number	Yes	Yes	Yes	Yes
Reported expiry date	Yes	Yes	Yes (as “Best be”)	Yes
Reported warnings	Yes	Yes	Yes	Yes
Legal statement	N.R.	N.R.	*These statements have not been evaluated by the Food and Drug Administration. This product is not intended for diagnosing, treating, curing, or preventing any disease.*	N.R.
Language of labeling	English and German	English and Italian	English	English, French, and Portuguese

^a^ Capsule.

^b^ Not reported.

^c^ Only reported the amount per serving (120 mg) and the serving size (two capsules) corresponding to an amount per capsule of 60 mg.

^d^ “*Each vegetarian capsule contains 400 mg of Mucuna pruriens standardized to 15% L-dopa*” corresponding to an amount per capsule of 60 mg.

^e^ Serving size: 2,000 mg *of Mucuna pruriens* extract standardized to 15% L-dopa corresponding to an amount per serving of 300 mg.

### 3.2 Trend of interest

**FIGURE 2:**
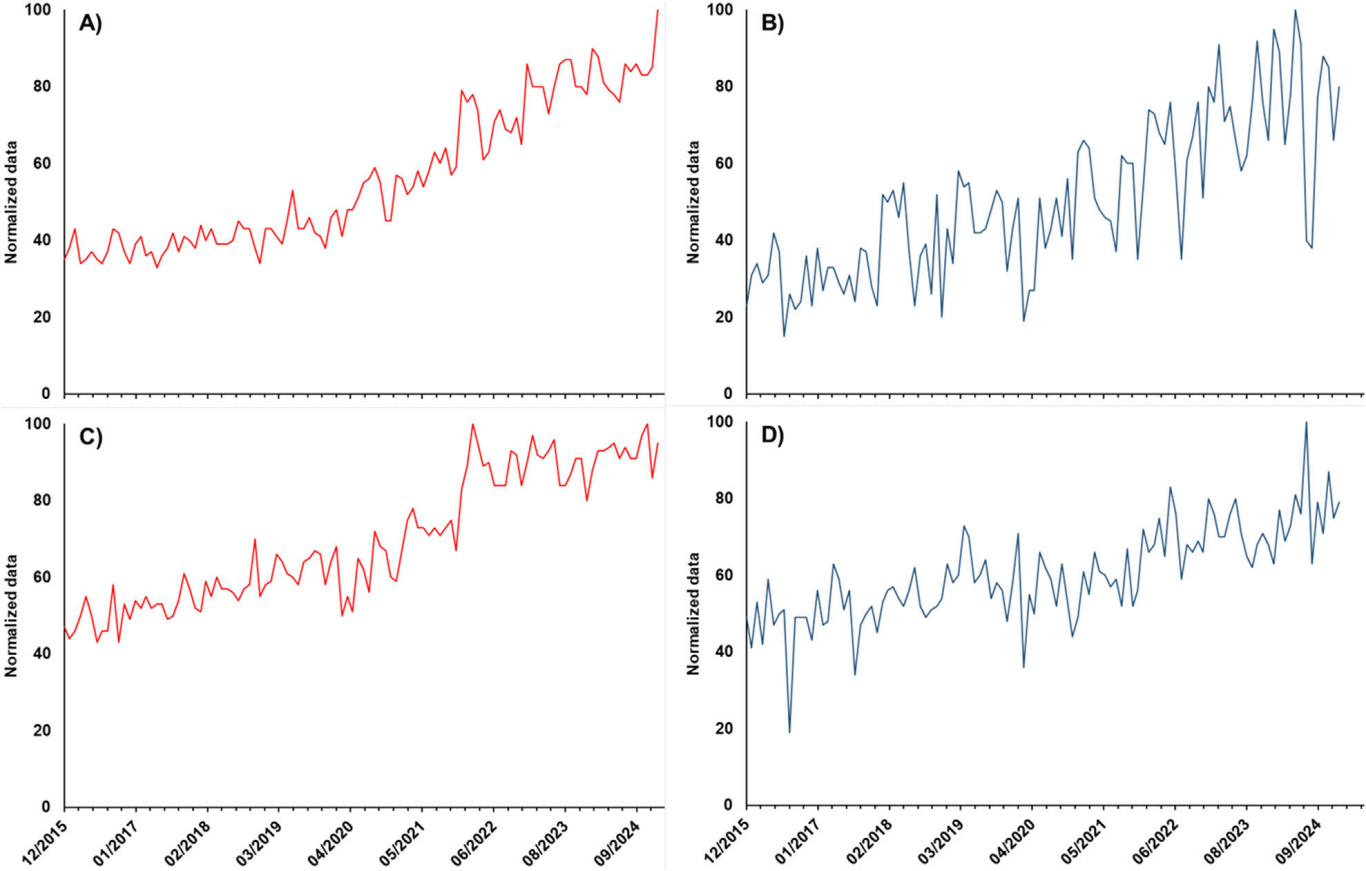
Trend of interest, obtained by the Google Trends application, for the search term “brain supplements” for the region “World” **(A)**, the combined search terms *“integratori memoria + integratori concentrazione”* for “Italy,” **(B)** and the search term “levodopa” for the “World” **(C)** and “Italy” **(D)** for “all categories” in the period from 01/12/2015 to 31/01/2025. Normalization was carried out on the higher search volume obtained in the considered period.

Google Trends is a tool to evaluate the interest in a single product or product categories based on the frequency of related search terms on Google. Figure 2 shows the trend of interest % for the term *“brain supplements”* for the region *“World”* (A) and for the combined search terms *“integratori memoria + integratori concentrazione”* for *“Italy”* (B) on the upper panels and the trend of interest % for the term “*levodopa”* in the World (C) and in Italy (D) on the lower panels in the period from 01/12/2015 to 31/01/2025. In all cases, an upward tendency can be observed, with a practically linear mode for “levodopa” in Italy and a more marked increase in 2022 in the world. It should be highlighted that this result, showing the overall trend, does not discriminate among searches for levodopa medicinal products and dietary supplements or other types of searches (for research or study purposes). Nevertheless, the data showed evidence of a general increase in interest for this molecule and the related market of products, which was also confirmed by the trend for *“brain supplements”* and the corresponding Italian terms that showed an evident increment starting from 2022.

### 3.3 Identification of L-dopa by mass spectrometry

**FIGURE 3:**
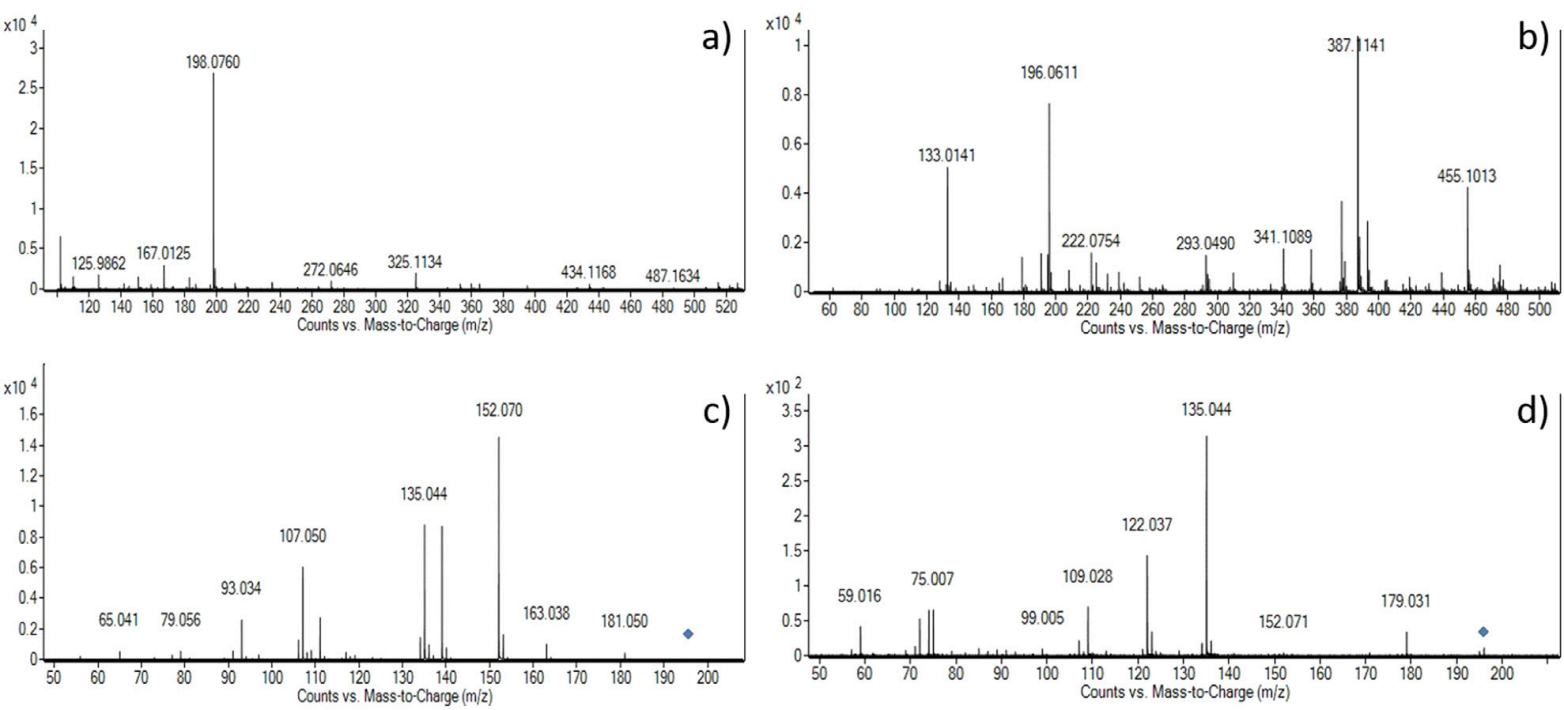
Mass spectra of the selected samples #4 **(a, c)** and #3 **(b, d)**. Upper panels depict mass spectra acquired in positive **(a)** and negative **(b)** modes, and the lower panels show the fragmentation pattern obtained at 20 V collision energy in positive **(c)** and negative **(d)** modes, respectively. The blue diamonds in panels c and d are the fragmented molecular ions (M + H)^+^ = 198.0760 and (M-H)^-^ = 196.0611, respectively.

The presence of L-dopa was confirmed in all samples by LC–MS–Q-TOF analysis, as depicted in Table 2. Extracted ion chromatogram (EIC) for the expected positive molecular ion at 198.0761 ([M + H]^+^) (see Supplementary Figure S1) showed correspondence of retention times compared to the L-dopa reference standard (Table 2). Moreover, accurate mass measurements obtained in positive mode by mass spectra extraction were in line with the theoretical mass, and the calculated error was <5 ppm in most cases. In sample #3, it was not possible to univocally assign the measured peak at 198.0895 to L-dopa; therefore, given the presence of an acidic moiety in the molecular structure of L-dopa, a negative mode analysis was tentatively used. A good correlation with the theoretical value of [M–H]^-^ with a calculated error on mass accuracy <4 ppm was obtained. In order to consider the compound identified and avoid false positive assignment, MS/MS fragmentation experiments were carried out, and the compound was considered identified if the accurate mass and relative abundance of the five most abundant fragments matched those of the reference standard ±0.01 Da and 20%, respectively. Accurate mass measurements of fragment ions, along with their relative abundance, matched those of the reference standard in both the positive and negative modes. In positive mode, fragments at 181.050 (loss of -NH_2_) and 152.070 (loss of -CO_2_H) were recorded as shown in Figure 3, further strengthening the proper identification of L-dopa.

**TABLE 2:** Retention time and accurate mass of molecular ions and fragments (in targeted mode) of the L-dopa peak in samples and in the reference standard obtained by LC–MS–Q-TOF analysis.

Sample #	RT^a^ (minutes)	m/z ([M + H]^+^) (Error, ppm^b^)	m/z ([M – H]^-^) (Error, ppm)	m/z of major fragment ions in positive mode (abundancies, %)	m/z of major fragment ions in negative mode (abundancies, %)
**1**	0.36	198.0761 (−0.18)	196.0612 (1.91)	**152.070 (base)**, 135.044 (61%), 139.039 (60%), 107.050 (40%), 111.044 (20%), 93.034 (17%), 163.040 (8%), and 140.041 (5%)	—
**2**	0.36	198.0760 (−0.49)	196.0612 (1.69)	**152.070 (base**), 135.044 (62%), 139.039 (62%), 107.050 (43%), 111.045 (20%), 93.034 (16%), 163.038 (7%), and 140.042 (5%)	—
**3**	0.36	198.0895^d^ (*67.65*)	196.0612 (3.87)	—	**135.044 (base)**, 122.037 (45%), 109.028 (22%), 74.024 (20%), 179.031 (10%), and 107.048 (7%)
**4**	0.36	198.0760 (0.43)	196.0606 (4.64)	**152.070 (base)**, 135.044 (61%), 139.039 (61%), 107.050 (42%), 111.044 (19%), 93.034 (18%), 163.038 (7%), and 140.042 (5%)	—
L-dopa Std^c^	0.36	198.0762 (−0.58)	196.0615 (1.86)	**152.071 (base)**, 135.044(72%), 139.039 (66%), 107.049 (51%), 111.043 (24%), 93.034 (16%), 163.039 (8%), and 140.041 (6%)	**135.044 (base)**, 122.036 (37%), 109.03(21%), 74.024 (18%). 179.039 (5%), and 107.0486 (5%)

^**a**^ Retention time of the chromatographic peak.

^**b**^ Parts per million.

^c^ Standard.

^d^ The positive ion was not univocally assigned to L-dopa, but the overall results allowed to univocally identify L-dopa.

Bold values represent the base peak.

No additional approved or unapproved pharmaceutical drug substances among those investigated from the PCDL database were found, including an exact mass search of carbidopa, entacapone, and benserazide, which are typically associated with synthetic levodopa in medicinal therapies. In addition, samples were screened for the presence of natural compounds by extracting the molecular ion chromatograms at (M+H)^+^ to rule out the use of synthetic levodopa as the only source of the active substance. Protonated species provisionally assigned to components such as arginine (molecular formula C_6_H_14_N_4_O_2_, extracted *m/z* 175.1195) and proline (molecular formula C_5_H_9_NO_2_, extracted *m/z* 116.0712) were detected, accounting for examples of endogenous metabolites of Fabaceae water and methanol extracts (Sruthi et al., 2023; Tesoro et al., 2024). LC–MS screening and the NMR profiles supported the presence of natural extracts along with L-dopa in all samples.

### 3.4 Identification and determination of the L-dopa content by NMR

The one- and two-dimensional ^1^H- and ^13^C-NMR spectra of the samples in methanol-d4 (MeOD) confirmed the presence of L-dopa in all the samples. The identification was carried out based on interpretation and comparison to the literature (Alnoman, 2024; Fernandez-Pastor et al., 2019; Talebpour et al., 2004). The three typical aromatic signals for ^1^H- and ^13^C-NMR were found at δ 6.73 (d)-117.0 and 6.72 (s)-117.0 and 6.60 (dd) −121.5; due to the large number of compounds in the methanolic solution, no other signals attributable to L-dopa were visible.

**FIGURE 4:**
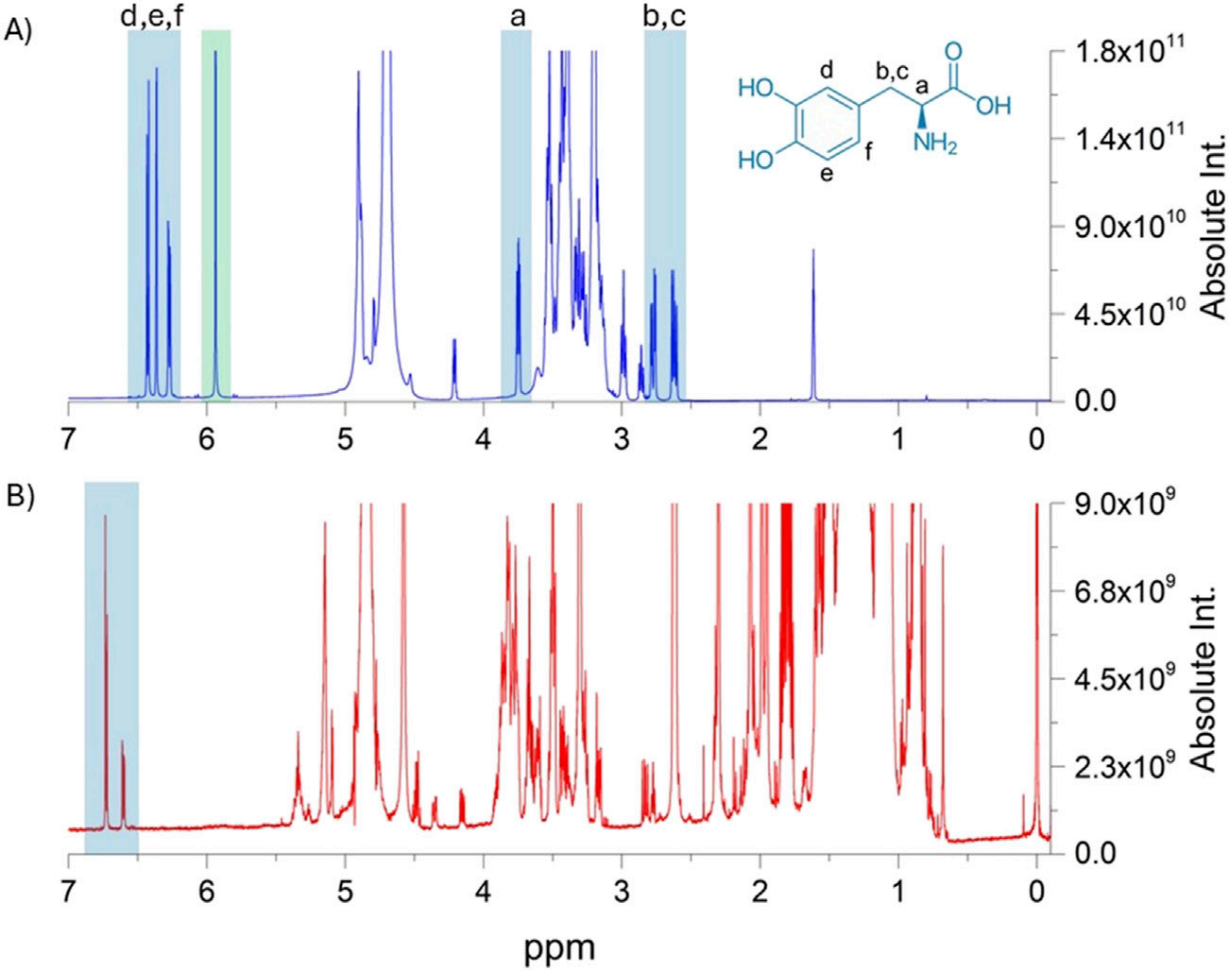
Comparison of the 1H-NMR spectra of the extraction procedures with the acid-d4/D2O mixture and MeOD in the upper **(A)** and lower **(B)** panels, respectively. In both profiles, involved L-dopa protons are highlighted in light blue boxes. The green box reports the signal of the internal standard, namely, maleic acid, which was used in q-NMR analysis.

Identification of L-dopa was also confirmed in the samples prepared for the quantification study. In this case, the extraction in the acetic acid-d4/D_2_O mixture proved to be more selective toward L-dopa, and all the protons and carbons of the molecule were detectable. The ^1^H-NMR spectra are shown in Figure 4, which includes the proton assignments for the same L-dopa sample dissolved in D_2_O/acetic acid-d4 and MeOD. In the ^1^H-NMR spectrum, signals ascribable to the aromatic protons d, e, and f were detected at δ 6.74 ppm (d, J = 2.1 Hz), 6.81 ppm (d, J = 8.0 Hz), and 6.65 ppm (dd, J = 8.0, 2.1 Hz), respectively. A more shielded signal at δ 3.84 ppm (dd, J = 8.0, 5.0 Hz) is attributable to proton *a*, whereas the b and c signals of the geminal protons in β with respect to the amine were identified in the two doublets at δ 3.07 pp (dd, J = 15.0, 8.0 Hz) and 2.91 ppm (dd, J = 15.0, 5.0 Hz). The protons a, b, and c in the spectrum with MeOD were not detectable.

In most cases, the one-dimensional proton spectra of plant extracts are highly populated and rich in signals, which may hinder identification and interfere with accurate quantification. The use of two-dimensional NMR ^1^H–^1^H and ^1^H–^13^C NMR experiments plays a fundamental role in the elucidation of molecular structures in mixtures without a database.

For q-NMR, L-dopa amounts were plotted against the corresponding powder weight, and the graphs were visually inspected for linearity. Bending of the linear curve at the higher concentration levels indicated that L-dopa had saturated the extraction solvent, which compromised the reliability of the quantification results (data not shown). In this case, the extraction process was repeated using larger volumes of the extraction solvent. A regression analysis was performed on the linear plots. The value of R^2^, the residuals (±2σ confidence interval), and the variance analysis (F < 0.05) confirmed a strong linear relationship between the L-dopa content and the powder amount, supporting quantitative extraction (Table 3). In conclusion, the accuracy was demonstrated by calculating the consistency of the results obtained at different concentrations in the linearity range. If extraction is non-quantitative, the L-dopa content result would not be proportional to the corresponding powder’s weight. This approach is widely accepted in q-NMR experiments. In each sample, the L-dopa content per capsule was calculated using at least four different concentrations, and experiments were performed in two different analytical sessions.

**TABLE 3:** Found content of L-dopa by q-NMR analysis and values of the regression line.

Sample	mg of L-dopa/capsule	%RSD^1^ (maximum acceptable limit)	Slope	Intercept	R^2^	F
1	101 ± 8	8.0% (13.33%)	0.179	0.379	0.995	2.05E-3
2	56 ± 6	10.0% (16.7%)	0.253	0.184	0.996	1.45E-3
3	73 ± 5	8.4% (8.80%)	0.257	0.1286	0.993	1.74E-5
4	89 ± 2	3.0% (10.50%)	0.190	0.036	0.997	7.38E-5

^1^ Relative standard deviation.

The content found in each experiment was normalized to the average weight of the capsules’ filling. For each sample, the RSD% (relative standard deviation) is reported in Table 3 with an evaluation of the maximum RSD% acceptable. Moreover, the two-tailed paired t-test with α = 0.05 confirmed that the results among the four sample were not statistically different. This shows the reproducibility and precision of the extraction and q-NMR methods. The calculated t value for each comparison within the sample dataset was less than the tabulated t value of 3.182. The uniformity of the mass of the single-dose preparation was tested for each sample according to European Pharmacopoeia 2.9.5. The results confirmed good homogeneity of the capsule content weight, thus supporting the use of 10 capsules for the determination of the average weight.

The labeled and found L-dopa contents are reported in Table 4. Comparison of the found and labeled amounts of L-dopa in the capsules evidenced that the declared content is confirmed for sample #1. Sample #2 was found to be approximately 50% underdosed, and sample #3 and #4 resulted were overdosed by approximately 120% and 150%, respectively. These findings should be considered in light of consumer safety, particularly for sample #4, where the actual daily intake of L-dopa—based on the suggested number of capsules per day—corresponded to approximately 445 mg.

**TABLE 4:** Found content of L-dopa and calculated percentage with respect to the reported content per capsule and with respect to the declared vegetal extract content. The actual daily intake was calculated based on the labeled serving size.

Samples	mg of L-dopa/capsule (found)	mg of L-dopa/capsule (declared or deduced)^a^	L-dopa/capsule (% found vs. declared or deduced)	% L-dopa/extract (declared)	Serving size^b^ in mg of L-dopa (suggested)	Real daily intake in mg of L-dopa based on serving size
1	101 ± 8	105	96%	24%	105	93–109
2	56 ± 6	117	48%	N.R^c^	117	50–62
3	73 ± 5	60^d^	122%	19%	120	136–156
4	89 ± 2	60^d^	148%	22%	300	435–455

^a^ This is the labeled content for samples 1 and 2. For samples 3 and 4, the content was deduced based on labeling, as reported in notes three and four of Table 1.

^b^ The serving size is the suggested intake per day.

^c^ Extract content not reported.

^d^ The content per capsule is not declared. The mg of L-dopa/capsule was deduced according to notes three and four of Table 1.

Finally, the percentages of L-dopa in the vegetal extracts were calculated considering the labeled content of the extract per capsule. The results, reported in Table 4, evidenced a percentage of 24% for sample # 1, matching the declared (25%), and percentages of 19% and 22% instead of the labeled 15% for samples # 3 and # 4, respectively. For sample # 2, the content of L-dopa reported was 98%; however, no information was present on the extract content. Therefore, the calculation was not possible. The only sample that showed an L-dopa content nearly equal to the declared amount (both in terms of content per capsule and the reported extract percentage) was sample # 1, which was also the only containing the *Vicia Faba* extract.

## 4 Discussion

In the present work, botanical preparations proposed as L-dopa dietary supplements and marketed online in Italy were analyzed, and despite the limited number of samples, the results evidenced many different quality issues.

Three samples analyzed in this study contained an extract from a plant that is not allowed in dietary supplements in Italy (*M. pruriens*) (Ministero della Salute, 2018); L-dopa is not included in the list of nutrients and other substances with a nutritional or physiological effect permitted in food supplements in Italy (Ministero della Salute, 2022b), making the samples illegal for the Italian market. Moreover, this plant is also not authorized as a novel food in Europe and is reported in the compendium of botanicals prepared by the European Food Safety Authority, a database of botanicals that are reported to contain naturally occurring substances of possible concern for human health when present in food (EFSA, 2012). The extract employed in the fourth sample (#1) originated from a plant that is, in principle, allowed in Italy for botanical preparations (*V. faba*); however, there was no clear indication of the part of the plant used, hindering the univocal identification of the extract source that may be subject to cross-contamination between different parts of the plant, with a possible consequence on the quality and safety of the extract. More importantly, all the samples claimed to contain a standardized L-dopa extract with levels ranging from 15% to 98%. It should be noted that the natural content of L-dopa in *V. faba* is < 1%–3% (Tesoro et al., 2024; Topal and Bozoğlu, 2016), whereas the levels in *Mucuna* seeds that are conventionally used in Ayurvedic medicines can be assumed to be 1%–7% (Nishihara et al., 2005; Pulikkalpura et al., 2015; Soares et al., 2014). This indicates that the extracts are the results of technological processes focusing on the extraction and reintroduction at much higher levels of substances occurring at low levels in botanical components, with adjustments made by inert excipients or blending of batches (EFSA, 2009; European Pharmacopoeia, 2019). These practices deviate from the conventional and traditional processing method, thus raising concerns regarding the safe use of such products without prior authorization at the European regulatory level. The Italian authorities have clarified that plant preparations and extracts used in food supplements must have levels of active substances comparable to those in traditional methods to avoid falling under the Novel Food Regulation (Ministero della Salute, 2022a; Morán and Kilasoniya, 2024). More specifically, in the framework of the Novel Food Regulation, novel aspects of the production process should be characterized when it has not been used for food production within the EU before 15 May 1997. Therefore, a scientific assessment to guarantee the safety of the production process used in the manufacture of the product, including, e.g., information on raw materials and processing aids, processing steps and operational conditions, and safety assurance measures, should be carried out as part of the commercialization procedure (EFSA, 2009). The results presented in this study demonstrate that illicit L-dopa samples are circulating in Italy, which inherently pose risks to consumers. For instance, the most common adverse effects associated with the intake of *M. pruriens* include dyskinesias and other types of involuntary movements, nausea, vomiting, psychotic episodes, hallucinations, and paranoid ideas (AECOSAN, 2016). Furthermore, the Dutch National Institute for Public Health and the Environment (De Heer et al., 2024) provided evidence of health risks related to the consumption of dietary supplements containing extracts of *M. pruriens*, concluding that it is not possible to determine a safe dose for these extracts.

All samples were marketed by the producers for recreational use, claiming the nootropic effects of L-dopa, which is an active pharmaceutical substance. In bodybuilding forums, cocktails of different substances containing L-dopa at different dosages are claimed for physical stimulation and to enhance aggressiveness. In the same forums, it is reported that L-dopa can be combined with caffeine to increase dopaminergic stimulation and with epigallocatechin gallate and other nootropic molecules such as racetams, adrafinil, and taurine (original information obtained by the authors from Italian bodybuilding forums). Furthermore, the overdosage of levodopa supplements, along with the frequent concomitant intake of other supplements containing nootropic substances such as caffeine, which synergistically interacts with levodopa and shortens the latency and increases the magnitude of the response, could represent a serious health risk to consumers (Deleu et al., 2006; Yu et al., 2006). The analyzed samples did not contain other nootropic or drug substances; however, this result is likely due to the limited number of samples analyzed; these findings also do not allow for speculation on geographical market differences or evolving manufacturing practices. The adulteration of these products cannot be ruled out and remains a potential risk to consumers, which should be addressed in wider surveillance studies covering Europe and Italy (Blazewicz A. et al., 2025; Gaudiano et al, 2024a; Vanhee et al., 2025).

The visual analysis evidenced severe deficiencies in labeling. This point should be stressed because unclear labeling can mislead consumers and lead to potential dosing errors. Moreover, even if the samples were sold in Italy through retail websites targeting the Italian market and providing information in Italian on their webpages, labeling was in Italian in only one sample. Quantitative NMR results, reported in Table 4, showed that the content per capsule was practically equal to the reported one (#1) in one sample, was approximately 50% of that labeled (#2) in another sample, and was significantly overdosed (approximately 120% in sample #3 and approximately 150% in sample #4) for the remaining two samples. The inconsistencies in the level of active compounds are aligned with previous findings on similar products bought online (Cohen et al., 2022; Soumyanath et al., 2018). Misdosing and ambiguous labeling are typical signs of the illegal market production (Gaudiano et al, 2024a; Gaudiano et al, 2024b; Gaudiano et al., 2022), where the quality criteria are barely met due to possible failures in production (e.g., inadequate purification processes), batch-to-batch inconsistencies, lack of good agricultural practice (e.g., pesticide residues), unsuitable starting and raw materials, and chemical and microbial contamination, including the potential presence of highly genotoxic aflatoxins.

This multicolored picture indicates that there is no certainty on the real content of L-dopa in these products, and the consumer can be exposed to very high dosages. Considering the number of capsules reported as “serving size” on the label, the observed variability is even more striking: from one product to another, the actual daily intake ranges from 56 mg to 445 mg. The content of L-dopa in the samples was relatively low to induce any therapeutic effect if considered individually; however, the suggested serving size resulted in an L-dopa intake close to or, in one case, even more than double the therapeutic dose for patients starting therapies with medicines containing L-dopa for Parkinson’s disease (200 mg) (De Heer et al., 2024). This should be considered with caution since botanical preparations containing L-dopa can also be consumed by Parkinson’s patients as a complement to standard pharmacological therapy (Cohen et al., 2021; Kispotta et al., 2024; Soumyanath et al., 2018). Health threats may originate from the interference with therapies, with consequences of developing severe dopamine dysregulation and resulting in patients’ hospitalization (Lambea-Gil et al., 2021; Vanhee et al., 2025).

Additional data were also obtained from the analytical domain. The combination of high-resolution mass spectrometry and NMR used in this study has proven to be a useful tool to identify molecules even in the presence of complex mixtures. In this study, the complexity of the NMR spectra and the results of MS spectrometry suggest the presence of natural components, strengthening the hypothesis of the presence of a botanical extract in the samples, but it is not possible to indicate if the high content of L-dopa is only because of the extraction and concentration procedures or because of the additional presence of synthetic L-dopa. Moreover, the analytical methods used did not allow for the distinction between L-dopa and D-dopa, and we assumed that the detected molecule is the L-enantiomer based on the presumed natural origin of the extracts, as the D-enantiomer has not been found in dietary supplements containing *M. pruriens* (Hasegawa et al., 2013). This assumption can be considered a limitation of the study that would warrants further investigation, considering that a potential role for D-dopa in dopamine production has been proposed in the development of new therapeutic strategies for Parkinson’s disease (Kawazoe T. et al., 2007). Moreover, as observed for other illegal products, it can be assumed that even for the same product, the variability among different batches could be high. Quantitative-NMR is a validated and reliable method for quantifying active ingredients, even in highly complex mixtures such as plant extracts, provided that at least one proton signal of the targeted molecule can be integrated without interference from overlapping signals. The advantage of this technique is the possibility to quantify any molecule containing paramagnetic atoms without a reference standard of the targeted molecule, which is, in the case of illegal products, a very useful tool.

Finally, the results of the present study should also be evaluated considering the information on the trend of interest for the terms “levodopa” and “brain supplements,” as indicated by data from the Google Trends application. The search showed evidence of a general increase in interest in L-dopa in the studied time period, both worldwide and in Italy, with a specific search for these products as brain support. The market of food supplements has expanded in volume and variety over the last 20 years, confirming the belief that they are an important part of people’s diets worldwide (Djaoudene et al., 2023). Italy is the leading market in Europe, with a share of 26% of the total turnover, followed by Germany (19%) and France (15%). The most prevalent sales channels are pharmacies, which are 78% of the total, followed by large-scale retail trade (with an incidence of 7.7%) and para-pharmacies (7.6%). Online channels account for 6.9% (Integratori and Salute, 2024). We have previously reported that illegal markets are fostered by the increase in interest in specific items (Gaudiano et al, 2024a); therefore, tracking the popularity of certain topics and products promotes the adoption of targeted quality assessments to tackle the phenomenon.

## 5 Conclusion

The results of this study provide evidence of the presence of commercial products proposed as dietary supplements claiming to contain botanical extracts that are highly concentrated in L-dopa and of plants (*M. pruriens*) not allowed in these products on the Internet market, which are accessible from Italy. The results of the visual inspection of labeling showed ambiguity on the amount of L-dopa per capsule and the identity of the manufacturers and the language not being accessible to Italian consumers. The analytical findings showed the presence of L-dopa in all samples but in quantities that differed from those reported, resulting in dangerous overdoses when considering the suggested serving size. The limited number of samples of this study may undermine the transferability of the results on a wider base; however, research focused on specific geographic markets is still limited, raising the need for further studies engaged in understanding the nature and diffusion among users of certain practices in order to implement targeted regulatory measures. Despite the limited number of samples, the risk to the consumers was well-evidenced. Consumers may not have immediate access to information regarding the illegal use of *M. pruriens*, and the presence of these products in the online market encourages their use. Unclear labeling on the actual L-dopa content and the manufacturer’s identity blurs traceability. The amount of L-dopa found in the capsules does not correspond to the labeled dose, and most importantly, in one case, it was higher than the therapeutic dose for patients starting therapies with medicines containing levodopa for Parkinson’s disease.

These findings have significant implications for healthcare professionals who recommend food supplements as adjuvants in Parkinson’s therapy and for general consumers seeking dopaminergic enhancement. In particular, efforts should be made to ensure that transparent information on dietary supplements sold online is readily accessible to consumers.

## Data Availability

The original contributions presented in the study are publicly available. This data can be found here: 10.6084/m9.figshare.30294385.

## References

[B1] Adebowale Y. A. Adeyemi A. Oshodi A. A. (2005). Variability in the physicochemical, nutritional and antinutritional attributes of six mucuna species. Food Chem. 89, 37–48. 10.1016/j.foodchem.2004.01.084

[B2] AECOSAN (2016). Report of the scientific committee of the Spanish agency for consumer affairs, food safety and nutrition (AECOSAN) on the risk of the use of seeds of *Mucuna pruriens* in craft products AECOSAN-2016-005. Madrid: AECOSAN.

[B3] Alnoman R. (2024). Design of chiral acidic molecularly imprinted polymer for the enantioselective separation of (±)‐DOPA. J. Chem. Technol. Biotechnol. 99 (8), 1879–1888. 10.1002/jctb.7691

[B4] Aware C. Patil R. Gaikwad S. Yadav S. Bapat V. Jadhav J. (2017). Evaluation of l-dopa, proximate composition with *in vitro* anti-inflammatory and antioxidant activity of Mucuna macrocarpa beans: a future drug for parkinson treatment, 7, 1097–1106. 10.1016/j.apjtb.2017.10.012

[B5] Bhushan M. Akash S. Pettarusp W. (2023). Adverse effects of medications used to treat motor symptoms of Parkinson′s disease: a narrative review. Ann. Mov. Disord. 6, 45–57. 10.4103/aomd.aomd_37_22

[B6] Blazewicz A. Poplawska M. Daniszewska B. Piorunska K. Karynski M. (2025). Illegal and falsified medicines self-administrated in not approved post-cycle therapy after the cessation of anabolic-androgenic steroids - qualitative analysis. Front. Chem. 2025 13, 1536858. 10.3389/fchem.2025.1536858 40177353 PMC11962791

[B7] Boelens Keun J. T. Arnoldussen I. A. Vriend C. van de Rest O. (2021). Dietary approaches to improve efficacy and control side effects of levodopa therapy in parkinson's disease. A Syst. Rev. 12, 2265–2287. 10.1093/advances/nmab060 34113965 PMC8634393

[B8] Cohen P. A. Avula B. Wang Y. H. Zakharevich I. Khan I. (2021). Five unapproved drugs found in cognitive enhancement supplements. Neurol. Clin. Pract. 11, e303–e307. 10.1212/CPJ.0000000000000960 34484905 PMC8382366

[B9] Cohen P. A. Avula B. Katragunta K. Khan I. (2022). Levodopa content of *Mucuna pruriens* supplements in the NIH dietary supplement label database. JAMA Neurol. 79, 1085–1086. 10.1001/jamaneurol.2022.2184 35939305 PMC9361182

[B10] De Heer J. A. Buijtenhuijs D. de Wit-Bos L. (2024). RIVM risk assessment of herbal preparations containing seed extracts of *Mucuna pruriens* . RIVM Lett. Rep. 2024-0087. 10.21945/RIVM-2024-0087

[B11] Deleu D. Jacob P. Chand P. Sarre S. Colwell A. (2006). Effects of caffeine on levodopa pharmacokinetics and pharmacodynamics in parkinson disease. Neurology 67, 897–899. 10.1212/01.wnl.0000233916.57415.9d 16966563

[B12] Djaoudene O. Romano A. Bradai Y. D. Zebiri F. Ouchene A. Yousfi Y. (2023). A global overview of dietary supplements: regulation, market trends, usage during the COVID-19 pandemic, and health effects. Health Eff. 15, 3320. 10.3390/nu15153320 37571258 PMC10421343

[B13] EFSA (2012). Compendium of botanicals reported to contain naturally occurring substances of possible concern for human health when used in food and food supplements. EFSA J. 10 (5), 2663. 10.2903/j.efsa.2012.2663 PMC13151549 42112181

[B14] EFSAEFSA Scientific Committee (2009). Guidance on safety assessment of botanicals and botanical preparations intended for use as ingredients in food supplements. EFSA J. 7 (9), 1249. 10.2903/j.efsa.2009.1249

[B15] European Pharmacopoeia (2019). Herbal drug extracts. Ph.Eur 11.0 04/2019, 0765.

[B16] Fernandez-Pastor I. Luque-Muñoz A. Rivas F. Medina-O’Donnell M. Martinez A. Gonzalez-Maldonado R. (2019). Quantitative NMR analysis of L-dopa in seeds from two varieties of *Mucuna pruriens* . Phytochem. Anal. 30, 89–94. 10.1002/pca.2793 30216583

[B17] Gaudiano M. C. Bertocchi P. De Orsi D. Manna L. Antoniella E. Rodomonte A. (2022). A case of medicine in disguise: motion sickness patches sold as medical devices containing active pharmaceutical substances. Ann. Ist. Super. Sanita 58, 254–263. 10.4415/ANN_22_04_05 36511196

[B18] Gaudiano M. C. Aureli F. Manna L. Borioni A. Maccelli A. Raimondo M. (2024a). Illegal products containing selective androgen receptor modulators purchased online from Italy: health risks for consumers. Sex. Med. 12, qfae018. 10.1093/sexmed/qfae018 38560649 PMC10973938

[B19] Gaudiano M. C. Aureli F. Alfonsi R. Rodomonte A. L. Sestili I. Bartolomei M. (2024b). Falsificazionedeimedicinali: daicasistoriciainuovi trend del fenomeno in Le attività di contrastodell’IstitutoSuperiore di Sanità. Rapporti ISTISAN 24/15. Roma: Istituto Superiore di Sanità.

[B20] Google (2023). Basics of google trends. Available online at: https://newsinitiative.withgoogle.com/it-it/resources/trainings/basics-ofgoogle-trends/(Accessed January 15, 2024).

[B21] Hasegawa T. Ishii T. Takahashi K. Saijo M. Fukiwake T. Nagata T. (2011). Quantitative determination of L-dopa in dietary supplements containing *Mucuna pruriens* by high performance liquid chromatography. Sci. Annu. Rep. 60, 53–56.

[B22] Hasegawa T. Takahashi K. Fukiwake T. Saijo M. Motoki Y. (2013). Enantiomeric determination of DOPA in dietary supplements containing *Mucuna pruriens* by liquid chromatography/mass spectrometry. Shokuhin Eiseigaku Zasshi 54, 379–383. 10.3358/shokueishi.54.379 24190293

[B23] Hornykiewicz O. (2017). L-dopa. J. Park. Dis. 7, S3–S10. 10.3233/JPD-179004 28282813 PMC5345651

[B24] Integratori and Salute (2024). Integratori, un comparto in crescita. Available online at: https://www.integratoriebenessere.it/assemblea-annuale/#_ftnref3 (Accessed March 13, 2025).

[B25] Kaced A. Belkacemi L. Chemat S. Taibi N. Bensouici C. Boussebaa W. (2024). Assessment of L-dopa, bioactive molecules and antioxidant activities of the local Algerian legume tadelaght (Vigna mungo l.Hepper) extract. Food Biosci. 61, 104902. 10.1016/j.fbio.2024.104902

[B26] Katzung B. G. Farmacologia V. T. W. Clinica G. e (2024). Italia: Piccin.

[B27] Kawazoe T. Tsuge H. Imagawa T. Aki K. Kuramitsu S. Fukui K. (2007). Structural basis of D-dopa oxidation by D-amino acid oxidase: alternative pathway for dopamine biosynthesis. Biochem. Biophys. Res. Commun. 355, 385–391. 10.1016/j.bbrc.2007.01.181 17303072

[B28] Kispotta S. Das D. Prusty S. K. (2024). A recent update on drugs and alternative approaches for parkinsonism. Neuropeptides 104, 102415. 10.1016/j.npep.2024.102415 38402775

[B29] Lambea-Gil A. María-Ángeles R.-C. Horna-Cañete L. Horna-Cañete L. (2021). Levodopa-induced dyskinesias related to Vicia faba ingestion in a parkinson's disease patient. Neurol. India 69, 1878–1879. 10.4103/0028-3886.333436 34979720

[B30] Ministero della Salute. (2018). DM 10 agosto 2018 “Disciplina dell’impiego negli integratori alimentari di sostanze e preparati vegetali e successivi aggiornamenti”.

[B31] Ministero della Salute. (2022a). Nota ministeriale “Indicazioni sull’uso delle piante e delle loro parti negli integratori alimentari per garantire la sicurezza e tutela dei cittadini”.

[B32] Ministero della Salute (2022b). Altri nutrienti e altre sostanze ad effetto nutritivo o fisiologico. Available online at: https://www.salute.gov.it/portale/alimentiParticolariIntegratori/dettaglioContenutiAlimentiParticolariIntegratori.jsp?lingua=italiano&id=1423&area=Alimenti%20particolari%20e%20integratori&menu=integratori (Accessed March 20, 2025).

[B33] Modi K. P. Patel N. M. Goyal R. K. (2008). Estimation of L-dopa from *Mucuna pruriens* LINN and formulations containing M. pruriens by HPTLC method. Chem. Pharm. Bull. (Tokyo) 56, 357–359. 10.1248/cpb.56.357 18310948

[B34] Morán J. Kilasoniya A. (2024). Application of the “Novel Foods” regulation to botanicals in the european union. Laws 13, 10. 10.3390/laws13010010

[B35] Mouchaileh N. Hughes A. J. (2020). Pharmacological management of Parkinson’s disease in older people. J. Pharm. Pract. Res. 50, 445–454. 10.1002/jppr.1683

[B36] Nishihara E. Parvez M. M. Araya H. Kawashima S. Fujii Y. (2005). L-3-(3,4-Dihydroxyphenyl)alanine (L-dopa), an allelochemical exudedfrom velvetbean (*Mucuna pruriens*) roots. Plant Growth Regul. 45, 113–120. 10.1007/s10725-005-0610-x

[B37] Polanowska K. Łukasik R. M. Kuligowski M. Nowak J. (2019). Development of a sustainable, simple, and robust method for efficient L-dopa extraction. Molecules 24, 2325. 10.3390/molecules24122325 31238569 PMC6631483

[B38] Pulikkalpura H. Kurup R. Mathew P. J. Baby S. (2015). Levodopa in *Mucuna pruriens* and its degradation. Sci. Rep. 5, 11078. 10.1038/srep11078 26058043 PMC4460905

[B39] Rezak M. (2007). Current pharmacotherapeutic treatment options in parkinson's disease. Dis. Mon. 53, 214–222. 10.1016/j.disamonth.2007.05.002 17586328

[B40] Soares A. R. Marchiosi R. Siqueira-Soares R. d. C. Barbosa de Lima R. Dantas dos Santos W. Ferrarese-Filho O. (2014). The role of L-dopa in plants. Plant. Signal. Behav. 9, e28275. 10.4161/psb.28275 24598311 PMC4091518

[B41] Soumyanath A. Denne T. Hiller A. Ramachandran S. Shinto L. (2018). Analysis of levodopa content in commercial *Mucuna pruriens* products using high-performance liquid chromatography with fluorescence detection. J. Altern. Complement. Med. 24, 182–186. 10.1089/acm.2017.0054 28922612 PMC5808387

[B42] Sruthi D. Jagannathan A. Chandran A. B. Rao H. C. Y. Jayabaskaran C. (2023). Chromatography-mass spectrometry based chemical profiling of *Mucuna pruriens* (L.) DC. And its beneficial effect against hydrogen peroxide-induced oxidative stress in HEK 293T cells and breast cancer cells. S. Afr. J. Bot. 159, 85–97. 10.1016/j.sajb.2023.06.003

[B43] Talebpour Z. Haghgoo S. Shamsipur M. (2004). 1H nuclear magnetic resonance spectroscopy analysis for simultaneous determination of levodopa, carbidopa and methyldopa in human serum and pharmaceutical formulations. Anal. Chim. Acta 506 (1), 97–104. 10.1016/j.aca.2003.10.081

[B44] Tesoro C. Lelario F. Ciriello R. Bianco G. Di Capua A. Acquavia M. A. (2022). An overview of methods for L-dopa extraction and analytical determination in plant matrices. Separations 9, 224. 10.3390/separations9080224

[B45] Tesoro C. Lelario F. Ciriello R. Bianco G. Acquavia M. A. Montoro P. (2023). A validated LC–MS/MS method for quantitative determination of L‐dopa in Fagioli di Sarconi beans (*Phaseolus vulgaris* L.). J. Mass Spectrom. 58, e4952. 10.1002/jms.4952 37401097

[B46] Tesoro C. Lelario F. Piscitelli F. Di Capua A. Della Sala P. Montoro P. (2024). Vicia faba L. pod valves: a By-Product with high potential as an adjuvant in the treatment of parkinson’s disease. Molecules 29, 3943. 10.3390/molecules29163943 39203021 PMC11357479

[B47] Topal N. Bozoğlu H. (2016). Determination of L-dopa L-3, 4-dihydroxyphenylalanine content of some faba bean Vicia faba L. Genotypes 22, 145–151. 10.1501/Tarimbil_0000001376

[B48] Vanhee C. Deconinck E. George M. Hansen A. Hackl A. Wollein U. (2025). The occurrence of illicit smart drugs or nootropics in Europe and Australia and their associated dangers: results from a market surveillance study by 12 official medicines control laboratories. J. Xenobiot. 15 (3), 88. 10.3390/jox15030088 40558871 PMC12193813

[B49] Vora R. Joshi A. Joshi N. (2018). Comparison of L-dopa content in three species of genus mucuna by different extraction techniques. Ann. Plant Sci. 7, 1973–1977. 10.21746/aps.2018.7.1.10

[B50] Yu L. Schwarzschild M. A. Chen J. F. (2006). Cross-sensitization between caffeine- and L-dopa-induced behaviors in hemiparkinsonian mice. Neurosci. Lett. 393, 31–35. 10.1016/j.neulet.2005.09.036 16236444

[B51] Yumoto E. Yanagihara N. Asahina M. (2022). The simple and rapid quantification method for L-3,4-dihydroxyphenylalanine (L-dopa) from plant sprout using liquid chromatography-mass spectrometry. Plant. Biotechnol. (Tokyo) 39, 199–204. 10.5511/plantbiotechnology.21.1126a 35937524 PMC9300427

[B52] Zhao J. Wang M. Saroja S. G. Khan I. A. (2022). NMR technique and methodology in botanical health product analysis and quality control. J. Pharm. Biomed. Anal. 207, 114376. 10.1016/j.jpba.2021.114376 34656935

