# Supplementary figures and images for: Quality assessment of “naturally occurring” high-percentage L-dopa commercial products proposed as dietary supplements on the Internet: from labeling to analytical findings
Source: Front Chem. 2025 Oct 15;13:1597784. doi: 10.3389/fchem.2025.1597784 (PMC12568576; doi:10.3389/fchem.2025.1597784)

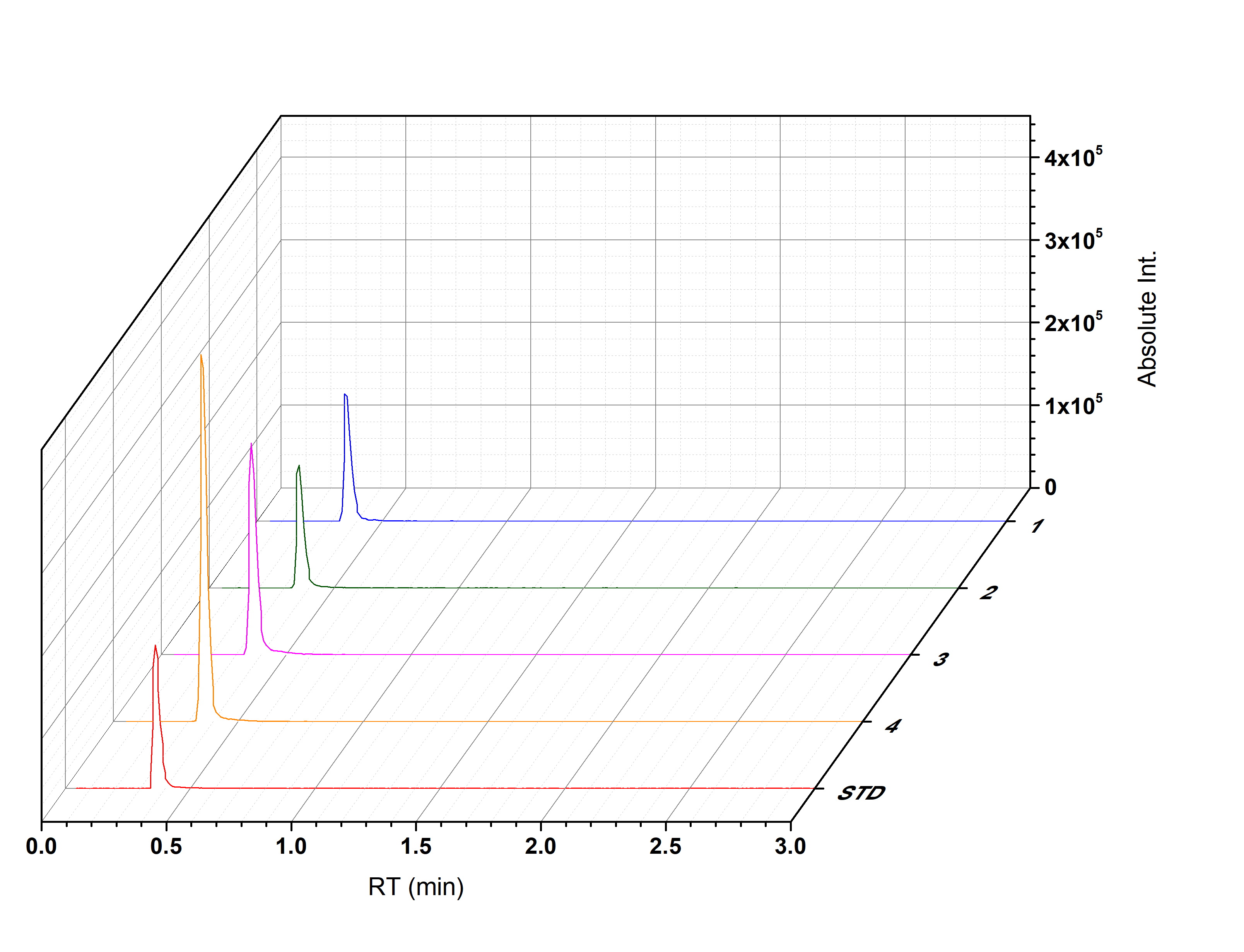

Supplement: Supplementary file 1 [file Image1.png]
